# Seeding Public Goods Is Essential for Maintaining Cooperation in *Pseudomonas aeruginosa*

**DOI:** 10.3389/fmicb.2019.02322

**Published:** 2019-10-09

**Authors:** Daniel Loarca, Dánae Díaz, Héctor Quezada, Ana Laura Guzmán-Ortiz, Abril Rebollar-Ruiz, Ana María Fernández Presas, Jimena Ramírez-Peris, Rafael Franco-Cendejas, Toshinari Maeda, Thomas K. Wood, Rodolfo García-Contreras

**Affiliations:** ^1^Departamento de Microbiología y Parasitología, Facultad de Medicina, Universidad Nacional Autónoma de México, Mexico City, Mexico; ^2^Laboratorio de Investigación en Inmunología y Proteómica, Hospital Infantil de México Federico Gómez, Mexico City, Mexico; ^3^Escuela Superior de Medicina, Instituto Politécnico Nacional, Mexico City, Mexico; ^4^Instituto Nacional de Psiquiatría Ramón de la Fuente Muñiz, Mexico City, Mexico; ^5^División de Infectología, Instituto Nacional de Rehabilitación Luis Guillermo Ibarra Ibarra, Mexico City, Mexico; ^6^Department of Biological Functions Engineering, Graduate School of Life Sciences and Systems Engineering, Kyushu Institute of Technology, Kitakyushu, Japan; ^7^Department of Chemical Engineering, The Pennsylvania State University, University Park, PA, United States

**Keywords:** social cheating, *Pseudomonas aeruginosa*, public goods, quorum sensing, tragedy of the commons

## Abstract

Quorum sensing in *Pseudomonas aeruginosa* controls the production of costly public goods such as exoproteases. This cooperative behavior is susceptible to social cheating by mutants that do not invest in the exoprotease production but assimilate the amino acids and peptides derived by the hydrolysis of proteins in the extracellular media. In sequential cultures with protein as the sole carbon source, these social cheaters are readily selected and often reach equilibrium with the exoprotease producers. Nevertheless, an excess of cheaters causes the collapse of population growth. In this work, using the reference strain PA14 and a clinical isolate from a burn patient, we demonstrate that the initial amount of public goods (exoprotease) that comes with the inoculum in each sequential culture is essential for maintaining population growth and that eliminating the exoprotease in the inoculum leads to rapid population collapse. Therefore, our results suggest that sequential washes should be combined with public good inhibitors to more effectively combat *P. aeruginosa* infections.

## Introduction

Quorum sensing (QS) in *P. aeruginosa* regulates the expression of several secreted products like exoproteases and siderophores, whose production is subject to social cheating by individuals that enjoy their benefit without contributing to their production (Diggle et al., 2007). In cultures using protein such as bovine serum albumin or casein as the sole carbon source, the expression of exoproteases is necessary to break the proteins down and then to assimilate the peptides and amino acids, derived from their hydrolysis. Hence, sequential culturing of *P. aeruginosa* in these conditions selects for the appearance of exoprotease-less mutants; these individuals are *lasR* mutants, i.e., they are quorum sensing blind mutants since they do not have the LasR response regulator, behave as social cheaters and have the potential to collapse the growth of the whole population by over exploitation of the cooperators (Sandoz et al., 2007). Those mutants are selected since LasR is the main receptor of QS, able to sense 3-oxo-C12-homoserine lactone and to activate the promoters of the genes of several exoproteases like the elastases *lasA* and *lasB* and also able to activate a second QS system mediated by RhlR which senses C4-homoserine lactone and also increases the expression of exoproteases (Castillo-Juárez et al., 2015).

Importantly, *lasR* mutants are also frequently isolated from chronic infections such as pulmonary infections of cystic fibrosis patients (Smith et al., 2006; D’Argenio et al., 2007; Hoffman et al., 2009) in combination with QS-proficient isolates (Wilder et al., 2009). Also, in experimental acute and chronic infections in mice, cheating *in vivo* using *lasR* and *lasI* mutants has been demonstrated (Rumbaugh et al., 2009).

Nevertheless, cheating also has negative consequences since it reduces fitness in stressful conditions; for example, QS-cheaters are more sensitive to oxidative, osmotic and heavy metal stress than cooperators (García-Contreras et al., 2015), and cooperators also have ways to protect themselves from over exploitation by producing policing metabolites like hydrogen cyanide and pyocyanin (Castañeda-Tamez et al., 2018), which limit the amount of cheating. Note that it was previously thought that hydrogen cyanide was a policing agent (Wang et al., 2015) but this has been shown to be unlikely (Smith et al., 2019). Hence, under standard culture conditions, population collapses are rare. For example, a recent study using strain SD-1 reported that no collapse was observed in 9 cultures after 30 daily passes in casein medium with NH_4_Cl as the nitrogen source and only in 1 of 9 cultures when casein was used both as the carbon and nitrogen source (Lai et al., 2018). Here, using both the reference strain PA14 and a clinical isolate from a burn patient (P279) in caseinate medium with NH_4_Cl, we show that (i) the initial amount of seeding of the public good exoproteases into each subculture is essential for maintaining growth and cooperation and that (ii) removing exoproteases by washing leads to a rapid population collapse in both strains, a finding that provides cues for understanding cheating dynamics and suggests that seeding exoprotease could be a relevant target for the design of antivirulence therapies.

## Materials and Methods

### Strains and Growth Conditions

Our experiments were performed with the laboratory strain *P. aeruginosa* PA14 (Liberati et al., 2006) and a clinical strain, P729, isolated from a burn patient from the collection of the Instituto Nacional de Rehabilitación Luis Guillermo Ibarra Ibarra in Mexico City. Growth was started from single colonies isolated from LB plates inoculated from glycerol stocks and was performed in LB medium at 37°C with 200 rpm of shaking for 16 h. Next, the precultures were used to initiate cultures with an initial turbidity of 0.05 at 600 nm in M9 medium with 0.25% sodium caseinate as the sole carbon source, and sequential subcultures were inoculated daily using either unwashed cells or cells washed with 1 mL of sterile 0.9% NaCl. In some experiments, exogenous exoprotease from *Streptomyces griseus* (P5147, SIGMA) was added to the washed cultures and in others, the M9 caseinate medium was supplemented with 0.025% of casamino acids.

### Characterization of Bacterial Populations

In order to quantify the proportion of protease-less individuals, aliquots of each sub culture were used to obtain single colonies in LB medium, and then those colonies were screened by growing them on M9 plates with casamino acids 0.25% and casein 0.5%, identifying the exoprotease producers by the formation of a clear hydrolytic halo surrounding the colony.

Caseinolitic activity was determined using azocasein (A2765, SIGMA) as the substrate according to procedures described in Nicodeme et al. (2005). Pyocyanin production as a second marker of QS activity was determined by extracting it from the supernatants with chloroform and then acidifying it with HCl and determinant the absorbance at 520 nm as described in Essar et al. (1990).

### Competition Experiments

Exoprotease-less, exoprotease producers, and the original P729 strain were used for competitions in M9 caseinate medium; the initial and final proportion of each strain were determined after 24 h of co-cultivation.

### Cheater Complementation

Exoprotease less individuals were complemented with the wild type *lasR* gene from wild-type PAO1 clonated in vector pUCP20 to form pMT1 (in which *lasR* is transcribed from its own promoter). pUCP20 was donated by Prof. Gloria Soberón Chávez from the Biomedical Research Institute at UNAM.

### Determination of Growth Rates and Mutation Frequencies

The growth rates of the PA14 wild-type washed and unwashed cultures in caseinate were determined by growth curves (recording O.D. at 600 nm each hour) and by obtaining the slope of the semi-logarithmic plot of time vs. O.D. 600 nm. The growth rate of the wild-type and cheaters in washed and unwashed cultures was estimated by growth curves combined with the estimation of cheater and wild-type proportions by plating on caseinate, every 2 h. In addition, lag phases were also estimated from the growth curves.

The mutation frequency of the wild-type PA14 and P729 strains and of one cheater from the unwashed cultures and one cheater from the washed cultures per strain was calculated by determining the colony forming units in the presence of 500 μg/mL of streptomycin relative to the colony forming units in the absence of antibiotic following the procedures described in Ciofu et al. (2005).

### Exoprotease Production Under Starving Conditions and Under the Effect of Carbonyl Cyanide *m*-Chlorophenyl Hydrazine

In order to determine the production of exoprotease under starvation conditions, strains were cultivated in LB medium for 16 h and then washed with sterile 0.9% NaCl, resuspended with M9 medium without a carbon source, incubated for 48 h at 37°C and 200 rpm and then the supernatant was collected and caseinolitic activity determined.

In order to determine the effect of carbonyl cyanide *m*-chlorophenyl hydrazine (CCCP) on the activity of exoprotease, the strains were grown in LB medium for 4.5 h and then CCCP dissolved in DMSO was added to a final concentration of 100 and 200 μM with an equivalent amount of DMSO used as a control, and the activity of exoprotease was assayed both before the CCCP addition and after 5 h of further incubation, using hide–remazol brilliant blue (H6268, SIGMA) as a substrate as described previously (Howe and Iglewski, 1984).

### Identification of the Exoprotease Produced Under Starving Conditions

In order to identify the proteases produced by the strains PA14 and P729, casein zymography was done as described (Caballero et al., 2001). To determine the zone of migration of casein exoproteases, in parallel, SDS-PAGE using the same conditions was performed, except casein was added to the gel, and the bands of interest were cut with a scalpel in pieces of approximately 1 mm^3^, and thoroughly washed with (i) 25 mM ammonium bicarbonate (ABC), 50% acetonitrile (ACN); (ii) 100% ACN; (iii) 50 mM ABC; and (iv) 100% ACN. 200 μL of 50 mM ABC were added to the dried gel pieces for reduction (10 mM DTT, 45 min, 56°C) and alkylation (20 mM Iodoacetamide, 30 min, room temperature in the dark). After washing the gel pieces, digestion were made with 25 ng/μL trypsin in the minimum volume of 25 mM ABC necessary to cover the gel pieces for 24 h. The peptide extraction was performed in two steps: the first extraction was made with 200 μL of 25% ACN, 1% FA during 15 min stirring every 5 min; the second extraction was made with 200 μL of 50% ACN, 1% FA for 30 min and then sonicated for 5 min. The two supernatants were pooled, dried and stored at −20°C. Just before injection to the nano HPLC system, samples were reconstituted in 15 μL of 0.1% FA, 5% ACN of which 3 μL were loaded into a Thermo UltiMate 3000 HPLC system using a pre-column/peptide trap Acclaim PepMap 100 C18 (300 μm × 1.5 cm), and a separation column Acclaim PepMap RSLC C18 (75 μm × 15 cm). Chromatographic runs were performed at a constant flow of 250 nL/min of a mix of 0.1% (v/v) FA in water (Buffer A), and 0.1% (v/v) FA in HPLC grade acetonitrile (Buffer B) in a linear gradient of 50 min from 1 to 50% B. Two analytical replicates were made for every sample. Electrospray ionization of the eluted peptides was performed with a CaptiveSpray source (Bruker) assisted by a flow of nitrogen boiled on acetonitrile (0.2 bar) and the mass spectra were acquired with a quadrupole time-of-flight mass spectrometer (Impact II, Bruker). Positive ions were analyzed over an m/z range of 50–2200. Before every six injections, calibration was performed with the ESI-TOF Tuning mix (Agilent). MS/MS fragmentation was performed for those ions with a signal higher than 500 counts applying a cycle time of 3 s and excluding +1 charged ions. Active exclusion was active after 2 spectra for 0.2 min, unless the intensity of the precursor was more than three times higher than in the previous scan. Collision energy depended on the precursor ion charge and mass (e.g., at 700 m/z, 33 eV and 27 eV for 2+ and 3+ ions respectively, whereas at 1100 m/z, 65 eV and 55 eV were used for 2+ and 3+ ions). Protein identifications were made processing the raw files with the DataAnalysis-otof-default script from the Bruker Compass Data Analysis software (version 4.2 SR2, Bruker), the ProteinScape software (version 3.1, Bruker) using Mascot 2.4.1 (Matrix Science): trypsin as the digestion enzyme, one miscleavage allowed, carbamidomethyl Cys as a fixed modification and oxidation on Met as variable modification. Monoisotopic peptide masses were searched with 10 ppm peptide mass tolerance and 0.05 Da fragment mass tolerance. FDR was set to 1% with the percolator and peptide decoy options active. The reviewed and unreviewed Swiss-Prot databases for *P. aeruginosa* were used. Proteins with Mascot scores >80, at least two peptides per protein and common to both analytical replicates were considered as successful identifications.

## Results

### Social Cheating in the Clinical Isolate

In order to evaluate if the isolate P729 was suitable for the experimental evolution experiments, first the production of exoproteases in this isolate and its growth in casein as the sole carbon source was confirmed. Then we performed serial cultures of the strain in casein and quantified the appearance of protease-less individuals by screening colonies on M9 plates with casamino acids 0.25% and casein 0.5%. As expected, this strain produced exoprotease-less mutants, and these mutants behaved as social cheaters within 24 h in competition experiments against the P729 parental strain with casein as sole carbon source (Supplementary Figures S1, S2).

### Washing the Precultures Promotes Population Collapses

**FIGURE 1:**
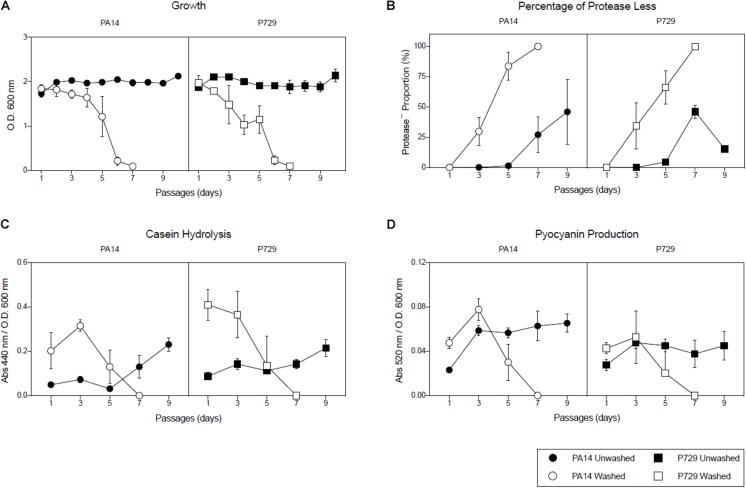
Washing promotes population collapses. **(A)** Growth of subcultures of the PA14 and P729 strains in casein after 24 h, **(B)**, percentage of protease less individuals of each subculture after 24 h, **(C)** caseinolitic activity of each subculture after 24 h, and **(D)** pyocyanin production in subcultures of PA14 and P729 strains in casein after 24 h, when cells were washed before daily inoculation (open symbols) or not washed (closed symbols). Results shown are the average of five independent cultures ± the standard deviation. Differences between washed and unwashed cultures were significant in a 2-way ANOVA *P* < 0.001.

In order to test the importance of seeding public goods (the amount of exoproteases added in each subculture with the inoculum), exoproteases were removed by washing the bacteria with isotonic saline solution prior to inoculating each culture, and (i) the percentage of protease-less individuals, (ii) the growth of each subculture after 24 h, (iii) pyocyanin production, and (iv) caseinolitic activity were recorded and compared with cultures inoculated without the washing step. Note that both caseinolitic activity and pyocyanin production are both tightly controlled by QS (Castillo-Juárez et al., 2015). As observed in Figure 1, population growth, pyocyanin production and caseinolitic activity collapsed after seven passages, in agreement with an abrupt increase in the cheater population. In contrast, collapses in the control group that lacked a washing step were rare as they were observed only after the 32nd passage for one out of four cultures of the strain P729 and after the 24th for one and 32nd passage for another PA14 culture out of four (Supplementary Figure S3).

To verify that the cheater selection occurred during the growth of the cultures and not during the previous washing step (for example if washing selectively affected the growth of the wild-type), mixtures of 50% wild-type and 50% cheaters were washed and immediately plated in order to determine their proportion, and, as expected, the proportion of cheaters remained unchanged after the washing (data not shown).

**FIGURE 2:**
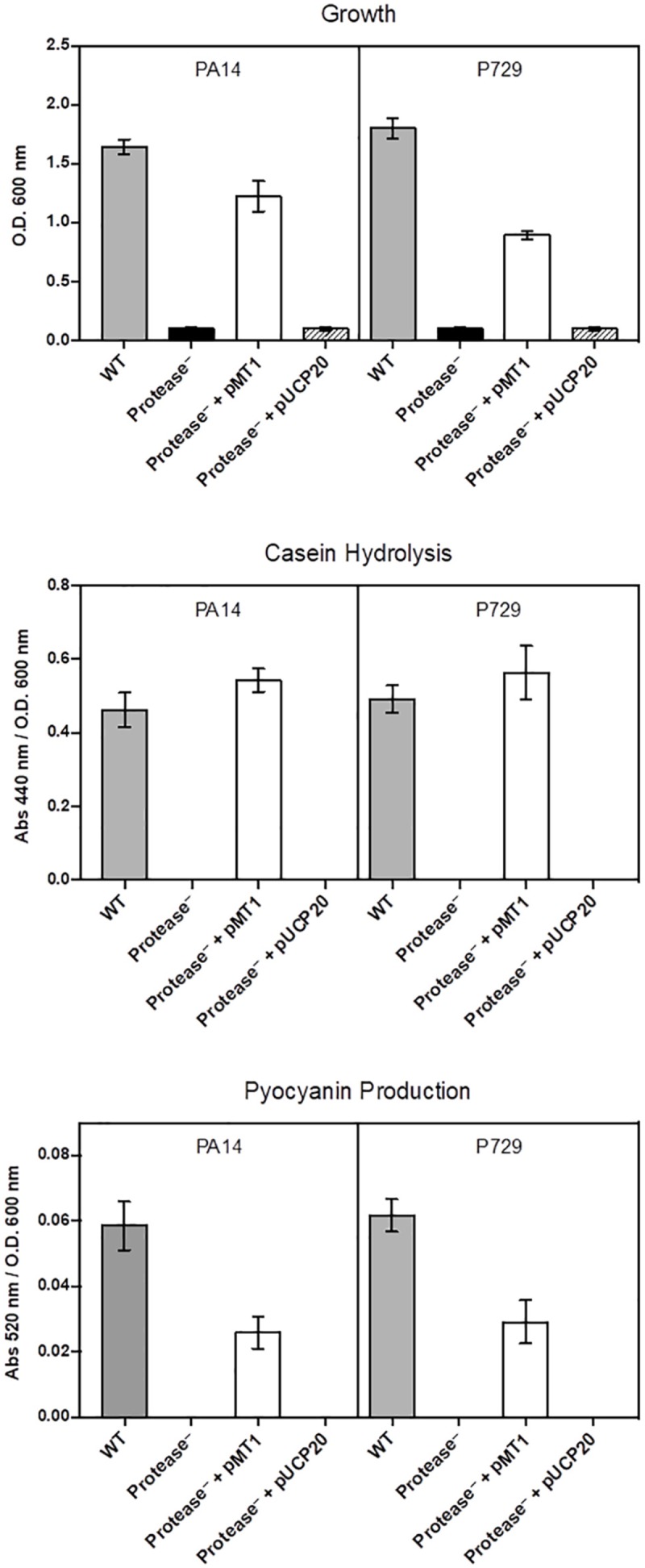
Complementation of cheaters with *lasR.* Results are from complementation of a protease-less PA14 and a protease-less P729 individual with PMT1 (pUCP20-*lasR*), controls carrying the vector alone (pUCP20) as well as a PA14 wild type. Phenotypes assayed include growth in caseinate as a sole carbon source, caseinolitic activity and pyocyanin production.

**FIGURE 3:**
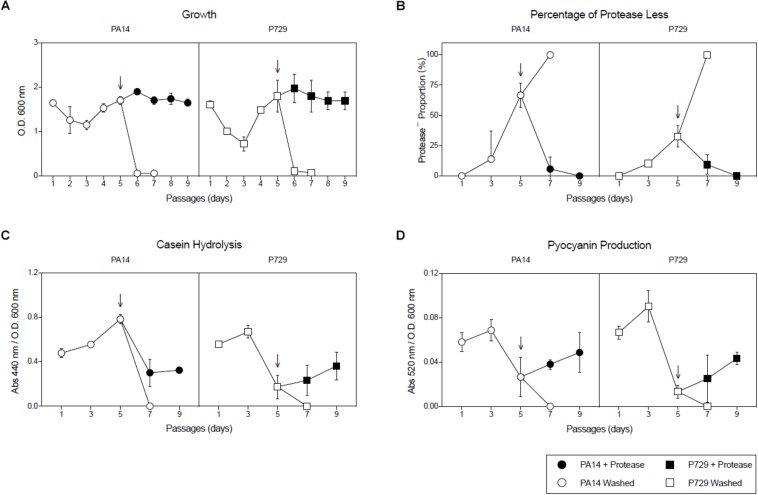
Adding exogenous protease suppresses growth collapses. **(A)** Growth of each PA14 and P729 subculture after 24 h, **(B)** percentage of protease-less individuals of each subculture after 24 h, **(C)** caseinolitic activity of each subculture after 24 h, and **(D)** pyocyanin production of each subculture after 24 h using washed inoculums without and with the addition of exogenous exoprotease during inoculation. Exoprotease was added at subculture five (indicated by arrows). Results shown are the average of three independent cultures ± the standard deviation. Differences between washed cultures with or without exogenous exoprotease were significant in a 2-way ANOVA *P* < 0.001.

In addition, cheaters were confirmed to be *lasR* mutants by complementation with the wild-type *lasR* gene (Figure 2). To confirm the population collapse was caused by removing the seeding exoproteases, an equivalent initial amount of exogenous exoprotease from *Streptomyces griseus* (P5147, SIGMA) was added together with the washed cells, and the culture growth did not collapse (Figure 3A). Instead, the proportion of cheaters decreased (Figure 3B), and casein hydrolysis/pyocyanin production recovered (Figures 3C,D). Moreover, adding a small amount of casamino acids (0.025%) to simulate the assimilable substrates produced by the casein hydrolysis also prevented the population collapse (Supplementary Figure S4).

### Mutation Frequencies and Growth

In order to elucidate the factors influencing the strong selection of the cheaters in the washed cultures, the mutation frequencies of the parental strains PA14 and P729 and of one cheater isolated from the unwashed cultures and one cheater isolated from the washed cultures per strain was determined. The results show no difference in the mutation frequencies of PA14 and the cheaters and a decrease in the mutation frequency of the cheaters derived from the P729 strain (Supplementary Table S1). Therefore we can rule out that increasing mutational frequency of the cheaters contributed to a higher appearance of cheaters in washed cultures. This result is in agreement to the observation of Sandoz and co-authors that determined no significant changes in exoprotease-less cheaters derived from strain PAO1 (Sandoz et al., 2007).

Regarding the growth rates, these were calculated in pure unwashed and washed cultures of the wild-type PA14 strains and in mixed cultures with cheaters and as expected, the growth rates of the cheaters were higher than the growth rates of the wild-type strain in both unwashed and washed cultures, and washing decreased both wild-type and cheater growth rates in mixed cultures, having no effect in the wild-type pure cultures. Interestingly, the presence of cheaters in unwashed cultures had no effect on the wild-type growth rate, but decreased it by 38% in washed cultures (Table 1), indicating washing allows cheaters to impose a higher energetic cost to the wild-type populations. Another important effect of washing was prolonging the length of lag phases in wild-type pure cultures (from 2 to 9 h) and in mixed cultures (from 3 to 12 h) (Table 1).

**TABLE 1:** Growth parameters.

	**unwashed**		**washed**	
**Strain**	**Lag phase**	**Growth rate**	**Lag phase**	**Growth rate**
Wild-type	2 h	0.246 ± 0.07	9 h	0.29 ± 0.021
Wild type (in the presence of cheater)	3 h	0.241 ± 0.038	12 h	0.18 ± 0.015
Cheater	3 h	0.365 ± 0.045	12 h	0.27 ± 0.015

*The growth rates of the PA14 wild-type washed and unwashed cultures in caseinate were determined by growth curves (recording O.D. at 600 nm each hour) and by obtaining the slope of the semi-logarithmic plot of time vs. O.D. 600 nm. The growth rate of the wild-type and cheaters in washed and unwashed cultures was estimated by growth curves combined with the estimation of cheater and wild-type proportions by plating on caseinate, every 2 h. In addition, lag phases (time in which no exponential growth was recorded) were also estimated from the growth curves. Results shown are the average of three independent experiments ± the standard deviation.*

### Exoprotease in the Cooperators Is Overproduced Under Starvation and Low Energy Conditions

**FIGURE 4:**
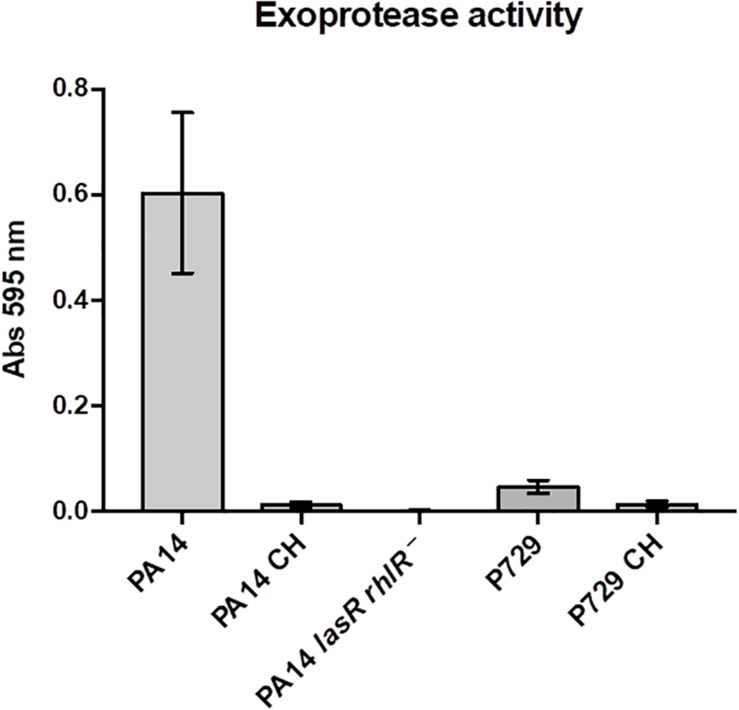
Exoprotease is produced after starvation only in QS proficient strains. Exoprotease is produced after starvation in M9 medium without a carbon source in strains PA14 and P729, but not in a *lasR rhlR* PA14 mutant, and very low activity was found in cheaters isolated from strains PA14 and P729. Results shown are the average ± SD of three independent cultures per strain.

Hence, we discovered that the presence of initial seeding exoproteases is essential to stabilize growth and cooperation and that their absence promotes population collapse. We also demonstrate that wild-type growth decreases in the presence of cheaters only in washed cultures, suggesting the energetic cost of producing exoprotease in that condition is higher than the cost in unwashed cultures (in the presence of seeding exoprotease). We hypothesized then that the amount of nutrients determines the level of exoproteases produced by the cooperators; i.e., the cooperators produce higher amounts of exoproteases as nutrients are depleted (hence their energy will be depleted and the amount of exoprotease they produce may not be enough to support the growth of a large proportion of cheaters). To test this, we cultured the bacteria to the stationary phase, washed them, and then resuspended them in M9 medium without a carbon source. We found that under these starvation conditions, exoprotease was produced by the wild type PA14 and P729 strains but not by cheaters derived from these strains or by a double PA14 *lasR rhlR* mutant; this strain was used as a control since it does not produce detectable exoprotease levels (García-Contreras et al., 2015; Figure 4). Moreover, the addition of the uncoupler CCCP at 100–200 μM that dissipates the proton motive force also promotes the production of exoprotease (Supplementary Figure S5A); these results corroborate an earlier finding demonstrating that lowering the proton motive force increases exoprotease production (Whooley and McLoughlin, 1983).

### The Exoprotease Produced Under Starvation Conditions Is LasB

The caseinolitic activity of the exoprotease produced by wild-type PA14 and P729 under starvation conditions was visualized by casein zymography using the bacterial supernatants of bacteria suspended in M9 salts during 48 h (Supplementary Figure S5B). The equivalent bands in an SDS-PAGE gel were cut and the proteins identified by mass spectrometry; we found that both PA14 and P729 produced elastase B (Supplementary Table S2), which is a QS-controlled exoprotease able to degrade casein. Moreover, the same result was obtained with another reference strain, PA01 (data not shown).

## Discussion

Our findings demonstrate that the presence of a small amount of public goods or resources for supporting initial growth are essential to stabilize cooperation in populations that include social cheaters that grow faster than the wild-type (Table 1), which is in agreement with the fact that *lasR* mutants assimilate and grow better in several amino acids as a carbon source (D’Argenio et al., 2007) and hence removing the seeding exoprotease strongly promotes population collapses. Taken together, the evidence presented here suggests that the initial amount of exoprotease seeding in each subculture allows an initial replication of wild-type producers that eventually is enough to produce additional exoprotease able to sustain their own growth and the growth of cheaters, and that the elimination of the exoprotease by washing stimulates the production of LasB protease by the wild-type individuals causing a higher energetic cost, increasing the length of the lag phase and decreasing the wild-type growth rate. However, the amount of *de novo* LasB that the wild-type strain can produce in the absence of seeding exoprotease is insufficient to allow the growth of the whole population, promoting the population collapse.

We hypothesize that this phenomenon may not be limited to exoproteases but perhaps applies to other public goods like the siderophore pyoverdine whose production also increases under conditions in which it is sequestered by gallium nitrate (Ross-Gillespie et al., 2014). This sequestration is equivalent to pyoverdine being utilized by non-pyoverdine producer cheaters, and may be an important factor for maintaining cooperation for environmental populations as well as for infections.

Our results are germane since, in clinical settings, cheating mutants (*lasR* and non-pyoverdine producers) are often found (Smith et al., 2006; D’Argenio et al., 2007; Hoffman et al., 2009; Wang et al., 2017), and antibiotic treatments in cystic fibrosis patients decrease the *P. aeruginosa* population significantly but often do not eliminate it, allowing recovery and re-colonization (Blanchard et al., 2017). Our findings suggest that simple actions like sequential washes, for example in burned patients infected with *P. aeruginosa* or bronchoalveolar lavages in infected cystic fibrosis patients alone and in combination with compounds that inhibit public goods, like iron chelators that target both metallo-exoproteases and siderophores, or more specific exoprotease inhibitors targeting LasB such as *N*-mercaptoacetyl-Phe-Tyr-amide (Cathcart et al., 2011) and gallium nitrate that sequesters siderophores (Ross-Gillespie et al., 2014), may help to avoid the re-growth of virulent public goods producing strains and hence may be a new strategy to combat *P. aeruginosa* infections and deserve further studies in animal infection models.

## Data Availability Statement

The datasets generated for this study are available on request to the corresponding author.

## Ethics Statement

The *Pseudomonas aeruginosa* clinical strain used in this study was isolated as part of routine clinical hospital procedures to diagnose infection and hence ethical approval was not required, according to the Instituto Nacional de Rehabilitación Luis Guillermo Ibarra Ibarra ethical committee.

## Author Contributions

DL, DD, HQ, AG-O, AR-R, AP, JR-P, and RG-C performed the experiments. DL, RF-C, TM, TW, and RG-C designed the study. DL analyzed the data. RG-C and TW wrote the manuscript.

## Conflict of Interest

The authors declare that the research was conducted in the absence of any commercial or financial relationships that could be construed as a potential conflict of interest.
