# Supplementary material for: Seeding Public Goods Is Essential for Maintaining Cooperation in Pseudomonas aeruginosa
Source: Front Microbiol. 2019 Oct 9;10:2322. doi: 10.3389/fmicb.2019.02322 (PMC6794470; doi:10.3389/fmicb.2019.02322)
Supplement: Supplementary file 1 [file Table_1.DOCX]

**Supplementary table 1 Mutation frequencies.**

| **strain** | **Mutation frequency** |
| --- | --- |
| PA14 wild-type | 1.89 x10^-8^ ± 1.59 x10^-8^ |
| PA14 cheater (unwashed) | 3.22 x10^-8^ ± 3.1 x10^-8^ |
| PA14 cheater (washed) | 4.3 x10^-8^ ± 0.75 x10^-8^ |
| P729 wild-type | 11.7 x10^-8^ ± 9.32 x10^-8^ |
| P729 cheater (unwashed) | 0.28 x10^-8^ ± 0.2 x10^-8^ |
| P729 cheater (washed) | 1.09 x10^-8^ ± 0.84 x10^-8^ |

The mutation frequency of the wild-type PA14 and P729 strains and of one cheater from the unwashed cultures and one cheater from the washed cultures per strain was calculated by determining the colony forming units in the presence of 500 μg/mL of streptomycin relative to the colony forming units in the absence of antibiotic. Results shown are the average of 3 independent experiments ± the standard deviation.
