# Supplementary material for: Seeding Public Goods Is Essential for Maintaining Cooperation in Pseudomonas aeruginosa
Source: Front Microbiol. 2019 Oct 9;10:2322. doi: 10.3389/fmicb.2019.02322 (PMC6794470; doi:10.3389/fmicb.2019.02322)
Supplement: Supplementary file 2 [file Table_2.DOCX]

**Mass spectrometry-based protein identification**

**Table S2. Proteins identified in supernatants of PA14 and P729 suspended in M9 medium without carbon source.**

| **Proteins identified in supernatant from PA14** | | | |
| --- | --- | --- | --- |
| **Uniprot accession** | **Protein** | **Scores** | **#Peptides** |
| P14756 | **Elastase LasB** | 166.7 | 7 |
| Q9HVG6 | Uncharacterized protein | 157.8 | 7 |
| **Identified proteins supernatant from PA729** | | | |
| **Uniprot accession** | **Protein** | **Scores** | **#Peptides** |
| P09591 | Elongation factor Tu | 1652.9 | 27 |
| Q9I589 | Chitin-binding protein CbpD | 1159.2 | 14 |
| Q9I3C5 | Chaperone protein HtpG | 951.1 | 23 |
| P30718 | 60 kDa chaperonin | 940.8 | 18 |
| Q9HV43 | Chaperone protein DnaK | 851.6 | 19 |
| P53593 | Succinate--CoA ligase [ADP-forming] subunit beta | 732.3 | 15 |
| Q9I3D1 | Dihydrolipoyl dehydrogenase | 692.4 | 17 |
| P13794 | Outer membrane porin F | 672.2 | 9 |
| Q9I0L5 | Isocitrate dehydrogenase [NADP] | 497.7 | 20 |
| Q9I1H5 | Chitinase | 478.8 | 13 |
| Q9HT20 | ATP synthase subunit beta | 459.6 | 12 |
| Q9HT18 | ATP synthase subunit alpha | 442.0 | 15 |
| Q9HZQ8 | Aminopeptidase | 441.2 | 10 |
| Q9I2A8 | Acetyl-CoA acetyltransferase | 414.7 | 11 |
| Q9HTX3 | Probable binding protein component of ABC iron transporter PA5217 | 379.8 | 8 |
| P14756 | **Elastase LasB** | 366.3 | 10 |
| Q9HWD2 | Elongation factor G 1 | 330.8 | 9 |
| O30508 | Succinylornithine transaminase/acetylornithine aminotransferase | 326.4 | 8 |
| Q9HX91 | Uncharacterized protein PA3922 | 313.2 | 9 |
| Q9HTD7 | Aspartate ammonia-lyase | 290.8 | 8 |
| Q9HVN5 | Chaperone protein ClpB | 288.0 | 9 |
| O82851 | Elongation factor Ts | 286.4 | 7 |
| Q9I6Z3 | Alkyl hydroperoxide reductase subunit C | 251.7 | 9 |
| Q9HY81 | Probable peroxidase | 248.4 | 8 |
| Q59636 | Nucleoside diphosphate kinase | 228.6 | 5 |
| Q9HZ71 | 30S ribosomal protein S1 | 227.8 | 8 |
| Q9I402 | Probable binding protein component of ABC transporter | 193.1 | 9 |
| Q9I4Z4 | Peptidoglycan-associated lipoprotein | 183.5 | 4 |
| Q9I244 | Elongation factor G 2 | 162.2 | 5 |
| O52762 | Catalase | 149.9 | 5 |
| Q9HZ48 | Probable binding protein component of ABC sugar transporter | 134.8 | 6 |
| P00282 | Azurin | 126.0 | 3 |
| Q9I6J1 | Putrescine-binding periplasmic protein SpuD | 121.8 | 3 |
| P72151 | B-type flagellin | 119.3 | 3 |
| P37798 | Biotin carboxylase | 116.6 | 2 |
| Q9HTP2 | Probable aldehyde dehydrogenase | 114.1 | 5 |
| Q9I5U9 | Uncharacterized protein | 103.0 | 5 |
| O50177 | Succinylglutamate desuccinylase | 99.8 | 4 |
| Q9I659 | Probable ClpA/B protease ATP binding subunit | 97.2 | 4 |
| Q9I6J0 | Spermidine-binding periplasmic protein SpuE | 94.3 | 3 |

.
