# Supplementary material for: Seeding Public Goods Is Essential for Maintaining Cooperation in Pseudomonas aeruginosa
Source: Front Microbiol. 2019 Oct 9;10:2322. doi: 10.3389/fmicb.2019.02322 (PMC6794470; doi:10.3389/fmicb.2019.02322)
Supplement: Supplementary file 3 [file Data_sheet_1.docx]

**Figure S1A**

Figure S1A. Exoprotease production of P729 parental strain and 2 protease (+) A and B, 2 protease (-) individuals C and D, isolated from casein sequential cultures at pass 20. Results are the average of 3 independent experiments ± the standard deviation.

**Fig S1 B**

Figure S1B. Growth of P729 parental strain and 2 protease (+) A and B, 2 protease (-) individuals C and D, isolated from casein sequential cultures at pass 2 in casamino acids and casein as sole carbon sources. Results are the average of 3 independent experiments ± the standard deviation.

**Figure S2**

Figure S2. 24h competitions experiments of the protease + P729 strains (parental and A,B) vs those that are protease – (C, D), in casein as sole carbon source, initial proportion of the protease less and its final proportion are shown.

**Figure S3**

Growth of serial unwashed cultures in casein, demonstrate population collapses are rare and occur after at least 24 passages. Cells were passed to a new flask every 24 h.

**Figure S4**

Final growth of subcultures of the PA14 and P729 strains using washed inoculums in casein plus casamino acids, A) growth, B) percentage of protease-less individuals, C) pyocyanin and D) caseinolytic activity in subcultures of PA14 and PA729 strains in casein 0.25% plus casamino acids 0.025%, when cells were washed before inoculation.

**Figure S5A**

1. CCCP induces the production of exoprotease in the PA14 strain, CCCP was added at 4.5 h and exoprotease activity was determined prior its addition and at 9 h. The increase by the addition of CCCP at 100 and 200 μM is significant relative to the exoprotease values in the control and DMSO treated cultures (P < 0.05 in a two-tailed T-test).

**Figure S5B**

**
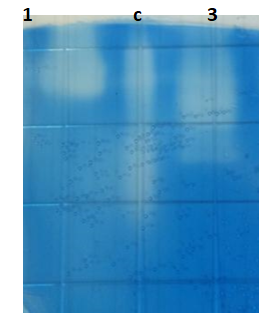
**

Casein Zymogram with supernatants from PA14 (1) and P729 (3) strains suspended in M9 medium without carbon source. C is a control protease from *Streptomyces griseus* (P5147, SIGMA).
